# The C3H zinc finger gene family in tartary buckwheat: genome-wide characterization, evolution, and a specialized role in salt stress response

**DOI:** 10.3389/fpls.2026.1865239

**Published:** 2026-06-22

**Authors:** Ling Zheng, Xueli Yang, Fei Wei, Jinbo Li

**Affiliations:** Department of Biology, Luoyang Normal University, Henan, Luoyang, China

**Keywords:** C3H zinc finger protein, drought stress, *Fagopyrum tataricum*, gene family, salt stress

## Abstract

Tartary buckwheat (*Fagopyrum tataricum*) is a medicinal and edible crop with remarkable adaptability to harsh environments. The C3H zinc finger protein family regulates plant growth and abiotic stress responses but remains uncharacterized in this species. Here, we identified 43 (*F. tataricum*) C3H (FtC3H) genes and classified them into six phylogenetic groups. Segmental duplication was the primary driver of family expansion, and all duplicated pairs underwent strong purifying selection (Ka/Ks < 1). All FtC3H proteins were predicted to localize to the nucleus. Promoters contained abundant hormone- and stress-related *cis*-elements, and FtC3H23 was predicted as a hub protein. Tissue-specific expression patterns indicated potential roles in flower, root, and stem development. Notably, most FtC3H genes were rapidly and strongly induced by salt stress; *FtC3H33* showed more than 100-fold upregulation, but exhibited minimal or suppressed expression under PEG-simulated drought. These findings reveal that the FtC3H family in Tartary buckwheat has evolved a specialized role in salt tolerance rather than general osmotic adaptation, with several members, particularly *FtC3H33*, as promising candidates for improving salt resistance.

## 1 Introduction

Buckwheat (*F. tataricum*) is an annual herbaceous plant belonging to the Polygonaceae family and *Fagopyrum* genus, commonly known as “clean intestine grass,” and is a medicinal and edible crop (Li et al., 2019). Buckwheat is rich in amino acids, resistant starch, vitamins, minerals, and bioactive flavonoid compounds. Numerous studies have shown that consuming buckwheat has therapeutic effects on diabetes, hypertension, and hyperlipidemia (Ren et al., 2014). Buckwheat exhibits strong ecological adaptability, tolerating poor soil and high-altitude cold conditions, and can thrive even in aluminum-rich acidic soils (Zhang et al., 2017). However, research indicates that drought stress and salt stress significantly impact various aspects of buckwheat, including seed germination (Li et al., 2022), aboveground agronomic traits (Zhao et al., 2019), and antioxidant enzyme activity (Lu et al., 2018). Therefore, identifying drought- and salt-resistant genes in buckwheat is crucial for ensuring its yield. As global temperatures rise, the impact of drought and other abiotic stresses on crop production is increasing. Buckwheat has a weak root system and is predominantly grown in rain-fed, high-altitude mountainous regions (Li et al., 2022), which are characterized by low rainfall, steep slopes, shallow soil layers, severe erosion, and limited water resources. Studies have shown that drought and salinization have become major factors affecting the emergence, yield, and quality of Tartary buckwheat, while also severely restricting its cultivation and industrial development (Hossain et al., 2020; Lu et al., 2026; Meng et al., 2022). Thus, the discovery and characterization of stress-resistance genes have become a top priority in Tartary buckwheat breeding programs.

Transcription factors play a crucial role in plant stress resistance. They are primarily categorized into three major families: the zinc finger protein (ZFP) family, the basic-loop-helix (bHLH) structure family, and the helix structure family unrelated to specific families. Zinc finger proteins (ZFPs) are named for their finger-like structures formed by the binding of one or more cysteine (Cys) residues and a histidine (His) residue with a zinc ion. Based on the number of cysteine and histidine residues and the spacing between them, ZFPs are classified into 10 main types: C2H2, C2HC, C2HC5, C2C2, C3H, C3HC4, C4, C4HC3, C6, and C8 (Kamaliyan and Clarke, 2024). The C3H gene family is an important subgroup within the zinc finger family, characterized by the presence of one or more C3H-type zinc finger motifs, composed of three cysteine residues and one histidine residue binding to a zinc ion. Further research has defined the consensus sequence of the C3H motif as C-X4-17-C-X4-6-C-X3-H, with C-X7-8-C-X5-C-X3-H being the most common motif in C3H proteins (Rameneni et al., 2018).

Increasing research demonstrates that the C3H gene family plays a significant role in regulating plant growth and development, as well as responses to biotic and abiotic stresses. In terms of growth and development regulation, in banana, the C3H zinc finger protein MaC3H33-like2 positively regulates fruit ripening by modulating genes in starch and cell wall degradation (Song et al., 2024). In Arabidopsis, the non-tandem C3H protein AtC3H12 negatively regulates seed germination and early seedling development (Seok et al., 2022). In transgenic soybeans, overexpression of *GmZF351* activates lipid biosynthesis genes, accelerating seed oil accumulation and increasing seed oil content (Li et al., 2017). Regarding abiotic stress regulation, the rice *TZF* gene *OsC3H10* has been shown to participate in drought resistance by increasing the expression of stress-related genes (Han et al., 2021). Overexpression of *GhC3H20* enhances salt stress tolerance in Arabidopsis and cotton through the ABA signal transduction pathway (Zhang et al., 2023). In tomato, the *SEC1-C3H39* module fine-tunes cold tolerance by mediating target mRNA degradation (Xu et al., 2023). In biotic stress resistance, Arabidopsis *C3H14* positively regulates defense against *Botrytis cinerea* in a *WRKY33*-dependent manner (Wang et al., 2020). In rice, phosphorylated *OsLIC* promotes *OsWRKY30* to confer resistance against bacterial blight and leaf streak diseases (Wang et al., 2022). In cotton, *GhZFP1*, as a nuclear protein, enhances transgenic plants’ tolerance to drought, salt, salicylic acid (SA) stress, and fungal diseases (Guo et al., 2022).

To date, genome-wide identification and characterization of the C3H gene family have been conducted in numerous plants, including *A. thaliana* (Wang et al., 2008), *Oryza sativa* (Wang et al., 2025), *Capsicum annuum* (Tang et al., 2023), *Solanum lycopersicum* (Li et al., 2025), and *Glycine max* (Alam et al., 2026). However, no comprehensive study has yet been reported on the C3H gene family members in buckwheat.

## 2 Methods

### 2.1 Identification of the C3H gene family in buckwheat

The buckwheat genomic data (cultivar Pinku1), including genomic sequences, protein data, and genome annotation files, were downloaded from the buckwheat genome database (https://www.mbkbase.org/Pinku1/). The Hidden Markov Model (HMM) file (PF00642) of the C3H conserved domain was downloaded from the Pfam database (https://www.ebi.ac.uk/interpro/entry/pfam/) (El-Gebali et al., 2019)and used to search the buckwheat genome protein file using the HMM Search tool in TBtools (V2.466) (Potter et al., 2018). Protein sequences with e-values less than or equal to e^-5^ were retained. Candidate genes were verified using the NCBI Conserved Domain Database (https://www.ncbi.nlm.nih.gov/Structure/bwrpsb/bwrpsb.cgi), and proteins lacking the C3H conserved domain and redundant sequences were removed (Marchler-Bauer et al., 2015).

The physicochemical properties of the buckwheat C3H family proteins were analyzed using the ExPASy online website (http://web.expasy.org/compute_pi/), obtaining data on amino acid length, molecular weight, isoelectric point, instability index, aliphatic index, and grand average of hydropathy (GRAVY). Subcellular localization of FtC3H proteins was predicted using Plant-mPLoc (http://www.csbio.sjtu.edu.cn/bioinf/plant-multi) with default parameters;only predictions with a reliability score ≥ 4 (on a scale of 1-5) were retained (Chou and Shen, 2010). For cross-validation, CELLO v2.5 (http://cello.life.nctu.edu.tw) was also used (Li et al., 2020). Predictions from both servers were compared, and only those showing consistent localization across the two platforms were considered reliable.

### 2.2 Phylogenetic tree construction of the C3H gene family

Amino acid sequence files of *A. thaliana* C3H proteins, grape C3H proteins, and tomato C3H proteins were downloaded from the Plant TFDB online website (https://planttfdb.gao-lab.org/). The MUSCLE function in MEGA 6 software was used to perform multiple sequence alignment of the amino acid sequences of buckwheat and the above three species. The Neighbor-Joining (NJ) method was used to construct the phylogenetic tree of the C3H family among the four species, generating an NWK file. The NWK file was uploaded to the iTOL online website, and various functions of the website were used to adjust and beautify the phylogenetic tree.

### 2.3 Gene structure and conserved motif analysis of the buckwheat C3H gene family

The protein sequence file of the buckwheat C3H gene family was imported into the MEME website (http://meme-suite.org/tools/meme). The maximum number of motifs was set to 10, with other parameters set to default values, to identify conserved motifs (Bailey et al., 2009). The protein sequence file of the buckwheat C3H gene family was uploaded to the NCBI CDD website to verify the position and number of C3H domains (Marchler-Bauer et al., 2015). Gene structure information, including intron-exon and UTR (untranslated region) distribution, was extracted from the buckwheat GFF file. The conserved motifs, domains, and gene structures of the buckwheat C3H gene family were visualized using the Gene Structure View function in TBtools (V2.466). The ClustalW function in MEGA 6 software was used to perform multiple sequence alignment of the amino acid sequences of buckwheat C3H proteins. The results of the multiple sequence alignment of buckwheat C3H proteins were presented using the GeneDoc software.

### 2.4 Gene duplication and synteny analysis

Based on the buckwheat reference genome and focusing on the C3H gene family, genome annotation data, comprising GFF files and protein sequences, served as the input for MCScanX to detect syntenic blocks, which were then graphically represented using circos. The “duplicate gene classifier” module of MCScanX was applied to categorize duplication types (Wang et al., 2012). In addition, Dupgen_finder was deployed to identify gene pairs corresponding to diverse duplication events, followed by the estimation of nonsynonymous (Ka) and synonymous (Ks) substitution rates via kaks_calculator (Zhang et al., 2006). Furthermore, to investigate the evolutionary origin of the FtC3H family, inter-species synteny analysis between Tartary buckwheat and *A. thaliana*, grape, and tomato was performed using the MCScanX pipeline within TBtools (V2.466).

### 2.5 Protein interaction and target gene prediction analysis

In the STRING database (version 12.0, https://string-db.org/), the protein sequences of the buckwheat C3H family members were uploaded to conduct node comparison. Relying on the *A. thaliana* protein interaction network, the relationships among buckwheat C3H family members were predicted. Only interactions with a combined confidence score ≥ 0.400 (medium confidence, the default threshold of STRING v12.0 for exploratory analysis) were retained. To focus on the internal network of buckwheat C3H proteins, interactions involving non−Ft proteins (e.g., Arabidopsis orthologs) were excluded from subsequent network visualization and hub analysis.

First, the *A. thaliana* C3H transcription factor binding profile (MA1756.2) was obtained from the JASPAR Plantae database (https://jaspar.elixir.no/search?q=&collection=CORE&tax_group=plants). After using TBtools (V2.466) to extract the 2000 bp promoter sequences upstream of all genes in the buckwheat genome, the online tool FIMO (https://meme-suite.org/meme/) was used to detect genes binding to C3H transcription factors (Chen et al., 2020). Subsequently, PFAM database was used for target gene domain prediction, and the OmicShare Tools online website (https://www.omicshare.com/tools) was used to perform GO (Gene Ontology) enrichment analysis on target genes to obtain the target gene prediction map.

### 2.6 Promoter analysis of the C3H genes

Using TB tools, the 2000 bp promoter sequences upstream of the transcription start sites of buckwheat C3H genes were obtained. The PlantCARE online website (http://bioinformatics.psb.ugent.be/webtools/plantcare/html/) was then used to analyze *cis*-acting elements. The Simple BioSequence Viewer function in TBtools (V2.466) was used to draw the *cis*-acting element map.

### 2.7 Transcriptome data processing and analysis

In this section, a publicly available dataset was selected from the CNCB database (https://www.cncb.ac.cn/), with the accession number PRJCA000407. Subsequently, the RNA-seq reads were aligned to the Tartary buckwheat reference genome (Zhang et al., 2017) utilizing Hisat2 (Kim et al., 2015). The aligned sequences, initially in SAM format, were then processed into sorted and indexed BAM files using Samtools (Li et al., 2009). Transcriptome assembly and abundance estimation were performed with StringTie (Pertea et al., 2015), which reconstructs transcript isoforms and quantifies their expression levels. Following this, raw read counts at the gene level were obtained through featureCounts (Liao et al., 2014) by assigning sequencing reads to annotated genomic features. Finally, the resulting count matrix was normalized to Fragments Per Kilobase of transcript per Million mapped reads (FPKM) using the R programming environment.

### 2.8 RNA extraction and qRT-PCR analysis

*F. tataricum* (cv. Xiqiao 1) seeds were sown in a peat moss-perlite-vermiculite mixture (3:1:1) and cultivated in a controlled growth chamber under a 16-h light/8-h dark cycle at 27°C and 22°C, respectively. At the three-week stage, seedlings were subjected to three separate treatments: 200 mM NaCl for salt stress, 30% PEG6000 for drought stress, and distilled water for the control group. Leaf tissues were collected at 0, 1, 3, 6, 12, and 24 h post-treatment, with three biological replicates per time point. All samples were immediately snap-frozen in liquid nitrogen and stored at −80 °C.

Total RNA was extracted using the Vazyme RNA isolation kit (RC411-C1), and cDNA was synthesized using the Vazyme reverse transcription kit (R223-01). Ten FtC3H genes were selected based on transcriptome data for qRT-PCR validation (primer sequences in **Supplementary Table 3**). Fifteen FtC3H genes were selected for qRT-PCR based on the presence of stress-related cis-elements (MBS, ABRE, TC-rich) in their promoters and detectable expression in leaves. Reactions were performed using ChamQ Universal SYBR qPCR Master Mix (Q711–02) with three biological replicates. The FtH3 gene was used as the internal reference, with primers FtH3_F (5′-GAAATTC GCAAGTA CCAGAAGAG-3′) and FtH3_R (5′-CCAACAAGGTA TGCCTCAGC-3′). Relative expression levels were calculated using the 2^−∆∆CT^ method (Livak and Schmittgen, 2001).

## 3 Results

### 3.1 Identification and analysis of the buckwheat C3H gene family

Identification results indicated the presence of 43 C3H transcription factors in the buckwheat genome; the basic physicochemical properties of the C3H gene family are presented in **Supplementary Table 1**. The length of C3H transcription factors ranged from 130 aa (FtC3H23) to 1008 aa (FtC3H15); the molecular weight of the proteins ranged from 15,276.37 Da (FtC3H23) to 114,686.64 Da (FtC3H15); and the theoretical isoelectric point (pI) varied from 5.43 (FtC3H5) to 9.38 (FtC3H19). Among them, 28 C3H transcription factors had an isoelectric point greater than 7, while 15 C3H transcription factors had a pI less than 7. The instability index results showed that only two C3H transcription factors (FtC3H15, FtC3H23) had instability indices lower than 40, classifying them as stable proteins; the remaining 41 C3H transcription factors were unstable proteins. The aliphatic index ranged from 40.71 (FtC3H30) to 85.18 (FtC3H15). The grand average of hydropathy (GRAVY) values were all negative, indicating that C3H transcription factors are hydrophilic proteins. It is speculated that these transcription factors can diffuse freely within the cell and rapidly respond to signals through hydrophilic modifications such as phosphorylation to achieve dynamic regulation. Subcellular localization results showed that all 43 C3H genes are located in the nucleus.

### 3.2 Phylogenetic analysis and gene structure of the C3H gene family

**Figure 1:**
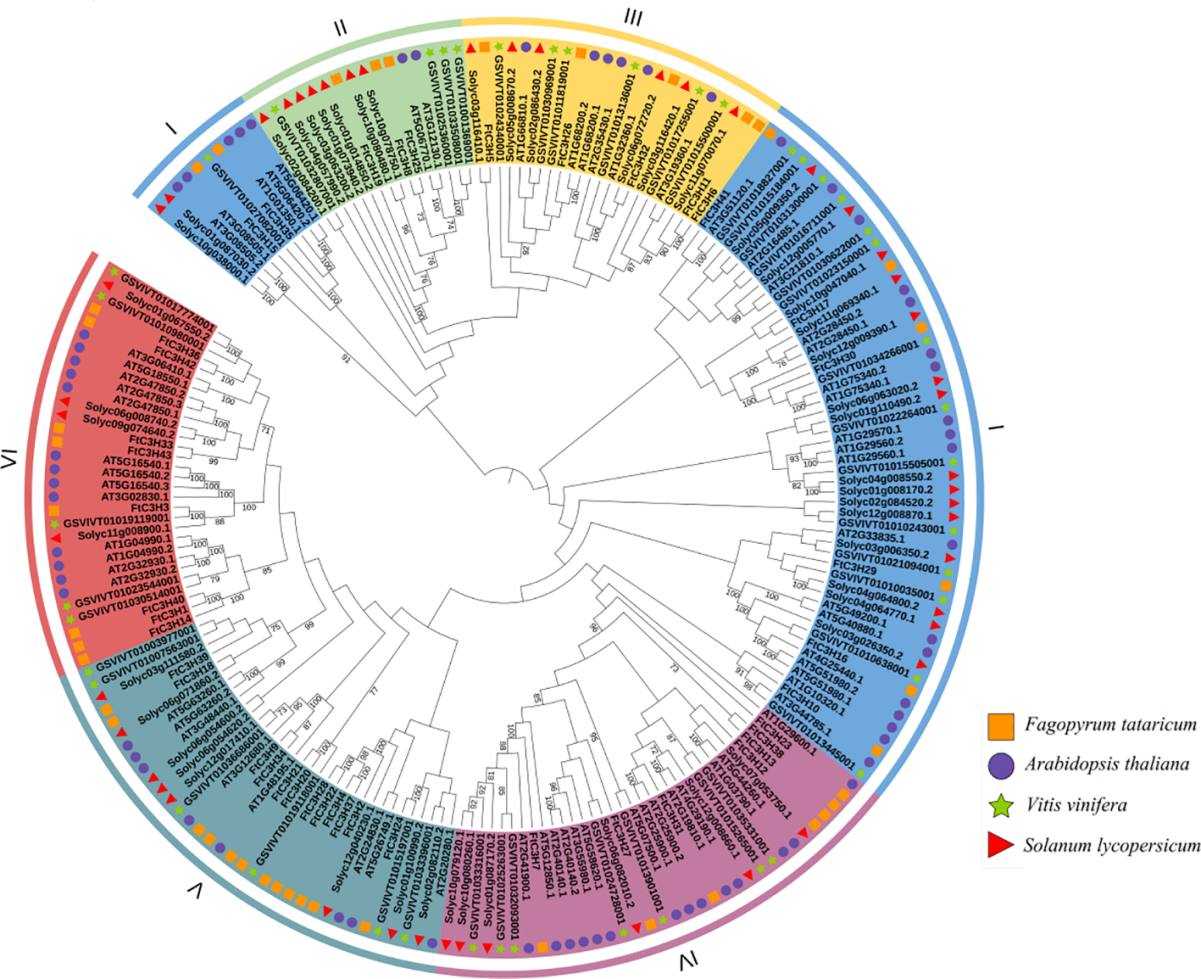
The Phylogenetic tree of C3H Gene Family in *F. tataricum, A. thaliana*, grape (*Vitis vinifera*), and tomato (*S. lycopersicum*).

To investigate the evolutionary relationships among C3H genes, we constructed a phylogenetic tree using full-length amino acid sequences from buckwheat, *A. thaliana*, grape, and tomato. Based on the classification of the Arabidopsis C3H family, the identified C3H genes were categorized into six distinct groups (Groups I–VI) (**Figure 1**). The FtC3H genes were distributed across these groups as follows: Group I (8 genes), Group II (3 genes), Group III (5 genes), Group IV (7 genes), Group V (12 genes), and Group VI (8 genes).

**Figure 2:**
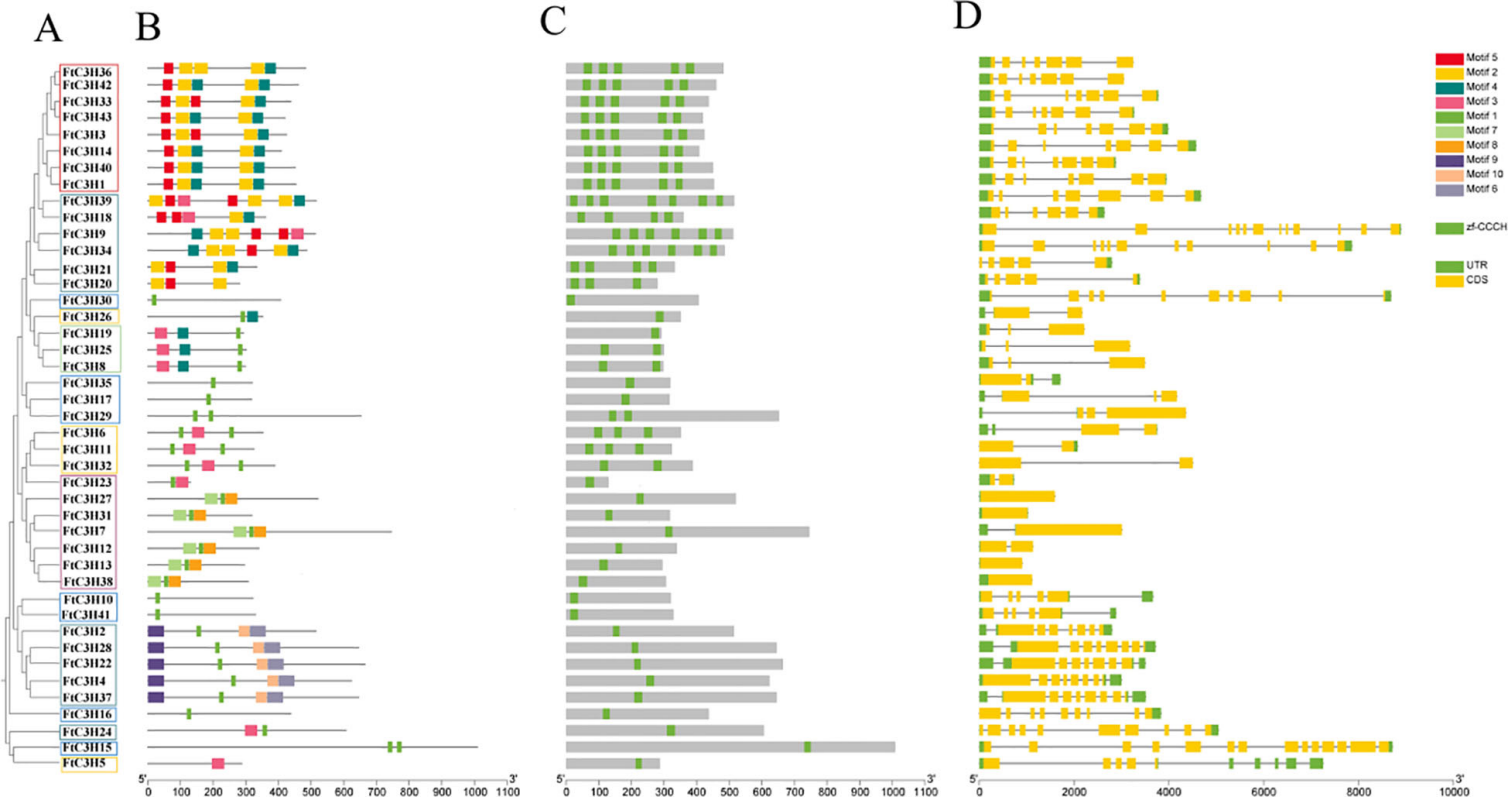
The Phylogenetic tree **(A)**, motifs **(B)**, domains **(C)** and gene structure **(D)** of C3H gene family in *F. tataricum*.

To further explore the structural diversity and conservation of these proteins, we constructed a phylogenetic tree of FtC3H genes (**Figure 2A**) and identified 10 conserved motifs (**Figure 2B**). Generally, genes within the same subgroup exhibited similar motif compositions, whereas distinct variations were observed between subgroups. For instance, members of Group II displayed a highly conserved pattern consisting of Motif 3, Motif 4, and Motif 1, suggesting strong functional conservation within this clade. Similarly, Group III was characterized primarily by Motif 3 and Motif 1, while Group IV members typically contained Motif 7, Motif 1, and Motif 8. In contrast, Group V members exhibited the most complex composition, containing a wide array of motifs (Motifs 1, 2, 4, 5, 6, 9, and 10), while Group VI members primarily possessed Motifs 2, 4, and 5. Additionally, domain analysis (**Figure 2C**) confirmed that every FtC3H member contains at least one C3H domain, with members of Groups V and VI possessing a higher density of 4 to 6 domains. As illustrated in **Supplementary Figure 1**, nearly every member of the buckwheat C3H family contains a C3H motif; however, variations in amino acid spacing and the number of C3H residues suggest that these differences may contribute to the functional specificity observed among different C3H members. Comparative analysis revealed that the C3H motif in buckwheat is predominantly of the C-X8-C-X5-C-X3-H type. This high prevalence suggests that this specific motif configuration may represent the ancestral form of C3H genes. Furthermore, the FtC3H proteins exhibit a high degree of conservation across the family, specifically characterized by the presence of a Glycine (Gly) residue between the second and third Cysteine (Cys) residues, and a Phenylalanine (Phe) residue between the third Cysteine (Cys) and the Histidine (His) residue.

We also analyzed the gene structures to determine exon-intron organization patterns (**Figure 2D**). The results revealed significant variation in genomic complexity across the family. Five members (11.63%)—specifically *FtC3H7*, *FtC3H13*, *FtC3H27*, *FtC3H31*, and *FtC3H38*—were intronless. While some genes contained only one (7 members) or two (5 members) introns, others were highly complex; for instance, *FtC3H15* contained 13 introns. The number of exons ranged from 1 to 14. Notably, distinct groups showed specific structural preferences: Group IV members had the simplest structure (mostly 1–2 exons), whereas Group V members had the highest average number of exons (ranging from 5 to 12). Groups II and VI showed uniform structures with 3 and 7 exons, respectively. Overall, Group IV exhibited the lowest average exon count, while Group V exhibited the highest.

### 3.3 Synteny analysis and selection pressure of the C3H gene family

**Figure 3:**
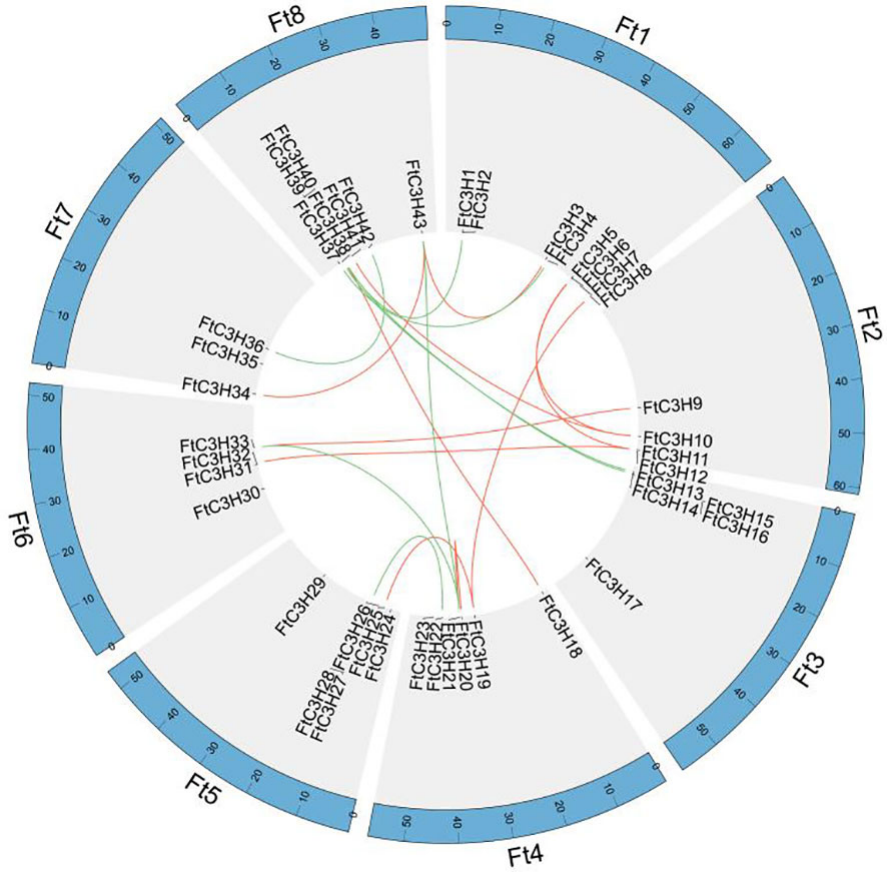
Synteny analysis of C3H genes in the buckwheat genome. The circular map displays the distribution of C3H genes across the eight pseudochromosomes (Ft1–Ft8). The gray inner circle represents the chromosomes, with gene loci marked by black boxes. Colored ribbons connecting gene pairs indicate duplication events: green lines represent whole-genome duplication (WGD) or segmental duplication events, while red lines represent transposed duplication events. The plot highlights the complex evolutionary history and expansion mechanisms of the C3H gene family.

To investigate the expansion mechanisms and evolutionary dynamics of the C3H gene family in buckwheat, we analyzed the syntenic relationships among the identified genes (**Figure 3**). The analysis reveals that the C3H family expansion was driven by both WGD and transposition. Several WGD-derived pairs (green links) were identified, such as the connection between *FtC3H1* and *FtC3H40*, and between *FtC3H4* and *FtC3H37*, suggesting that segmental duplications have played a significant role in the retention of these genes. Additionally, transposed duplications are widely distributed, involving genes such as *FtC3H10*, *FtC3H11*, and *FtC3H12* on chromosome Ft2. These results indicate that distinct duplication mechanisms have collectively contributed to the diversification and expansion of the C3H gene family in the buckwheat genome. Additionally, to investigate the origin and evolution of the C3H gene family in Tartary buckwheat, we analyzed the synteny of C3H genes among *F. tataricum*, *A. thaliana*, grape (*V. vinifera*), and tomato (*S. lycopersicum*) using TBtools. The results showed that Tartary buckwheat shares 16, 18, and 15 syntenic orthologous gene pairs with Arabidopsis, grape, and tomato, respectively (**Supplementary Figure 2**).

The non-synonymous (Ka), synonymous (Ks) substitution rates, and their ratios (Ka/Ks) for all collinear gene pairs identified in the syntenic analysis was also calculated. The Ka/Ks ratios for transposed duplication events ranged from 0.059 to 0.460, with the majority exhibiting ratios below 0.1. Similarly, the WGD/segmental duplication pairs displayed Ka/Ks ratios ranging from 0.079 to 0.235. The absence of Ka/Ks ratios greater than 1 suggests that the C3H gene family in buckwheat has primarily evolved under strong purifying selection (**Supplementary Table 2**). This evolutionary pressure acts to eliminate deleterious mutations and maintain the structural and functional stability of these transcription factors following duplication events.

### 3.4 Protein interaction and target gene prediction analysis of the buckwheat C3H gene family

**Figure 4:**
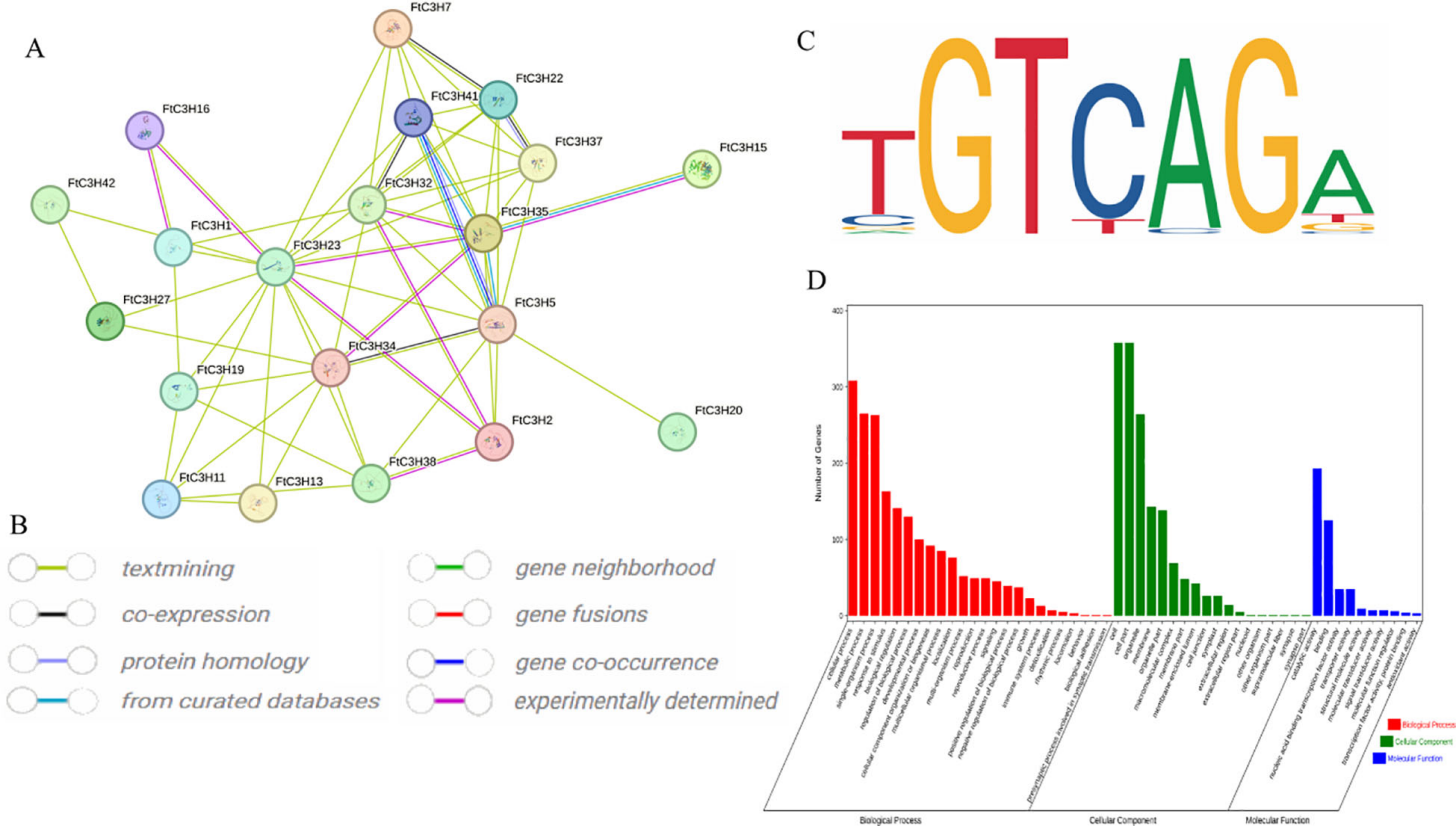
Protein interaction and target gene analysis of C3H gene family in *F. tataricum.* **(A)** Protein interaction network of FtC3H family members. **(B)** The meaning of different colored lines in the interaction network. **(C)** GRF9 (C3H) transcription factor binding profile. **(D)** GO enrichment results for target genes.

Leveraging the protein interaction network of *A. thaliana*, we predicted the potential protein-protein interactions (PPI) among buckwheat C3H family members using the STRING database (**Figures 4A, B**). The results indicated that 20 members of the FtC3H family are involved in potential interaction networks. Notably, FtC3H23 was identified as a potential hub protein, with 16 interaction partners among FtC3H family members. The combined STRING confidence scores of these interactions ranged from 0.639 to 0.897 (average 0.750 ± 0.069), and 14 of them (87.5%) were classified as high confidence (≥0.700). Following this, FtC3H32, FtC3H34, FtC3H35, and FtC3H5 exhibited extensive connectivity, interacting with 10, 9, 8, and 7 proteins, respectively. In contrast, *FtC3H15* and *FtC3H20* showed the fewest interactions, each connecting with only one partner (*FtC3H15* with *FtC3H35*, and *FtC3H20* with *FtC3H5*). Gene Ontology (GO) enrichment analysis of these interacting proteins revealed significant functional diversity, with enrichment in terms such as proton-transporting ATP synthase activity, rotational mechanism (GO:0046933), 3-oxoacyl-[acyl-carrier- protein] reductase (NADPH) activity (GO:0004316), glutaminase activity (GO:0004359), and heat shock protein binding (GO:0031072).

To further explore the regulatory networks, we identified a total of 5,147 potential target genes in the buckwheat genome based on the binding motif of the C3H12 transcription factor (MA1756.2). The matched sequence for these targets was identified as TGTCAGA (**Figure 4C**). GO enrichment analysis of these target genes was conducted across three categories (**Figure 4D**). In the Biological Process category, the target genes were primarily enriched in cellular process (GO:0009987), metabolic process (GO:0008152), and single-organism process (GO:0044699). In the Cellular Component category, the majority of targets were associated with cell (GO:0005623), cell part (GO:0044464), and organelle (GO:0043226). Finally, in the Molecular Function category, the target genes were mainly enriched in catalytic activity (GO:0003824) and binding (GO:0005488).

### 3.5 Cis-acting regulatory element analysis of the FtC3H gene family

To investigate the potential regulatory mechanisms of *FtC3H* genes, we analyzed the *cis*-acting regulatory elements in their promoter sequences. This analysis identified a total of 24 distinct types of *cis*-acting elements, categorized into three major functional groups: stress response, hormone regulation, and growth/development.

**Figure 5:**
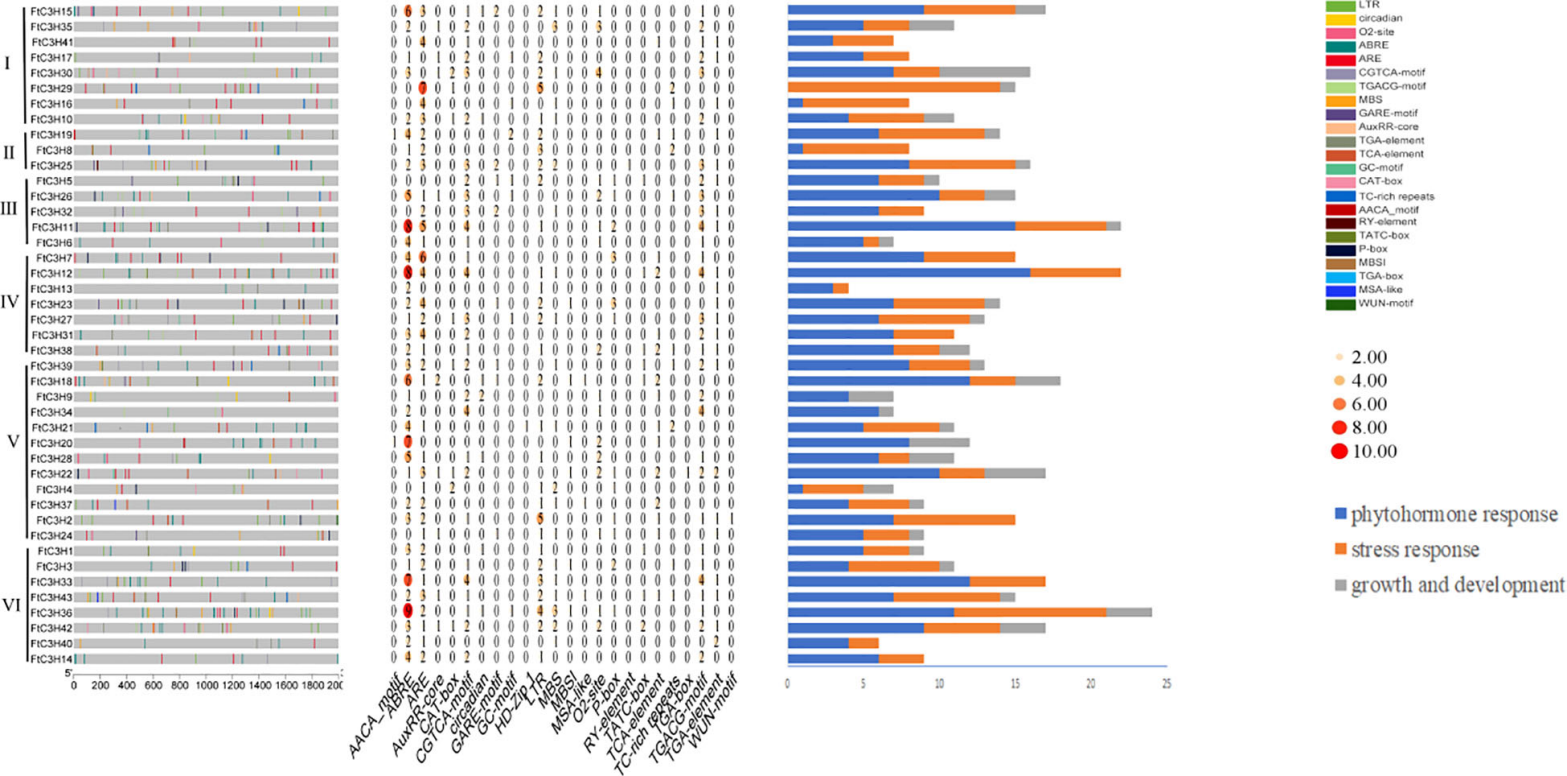
The *cis*-elements of C3H gene family in *F. tataricum.* Analysis of *cis*-acting elements in the promoter regions of FtC3H genes, numbers indicate the number of *cis*-acting elements, and histogram of *cis*-acting elements in each FtC3H genes.

As shown in **Figure 5**, the promoter regions harbor 10 types of hormone- responsive elements. These include gibberellin-responsive elements (P-box, GARE-motif, TATC-box), abscisic acid (ABA)-responsive elements (ABRE), salicylic acid-responsive elements (TCA-element), auxin-responsive elements (TGA-element, TGA-box, AuxRR-core), and methyl jasmonate-responsive elements (TGACG-motif, CGTCA-motif). Among these, the ABRE (abscisic acid response element) was the most abundant and widely distributed across the C3H members, suggesting a strong link to stress signaling pathways.

Furthermore, 8 types of elements associated with growth and development were identified, including MBSI, O2-site, MSA-like, circadian, CAT-box, RY-element, AACA_motif, and HD-Zip 1. Notably, the O2-site and CAT-box were frequently detected in the FtC3H promoters, implying that these genes are likely involved in various developmental processes. Finally, 6 types of stress-responsive elements were found, including the low-temperature responsive element (LTR), drought-responsive element (MBS), anaerobic induction element (ARE), and wound-responsive element (WUN-motif). The high prevalence of LTR and ARE elements in the FtC3H family suggests that these genes may play significant roles in conferring tolerance to low temperature and anaerobic stresses. The detailed distribution of these *cis*-elements across the three functional categories is summarized in **Supplementary Table 4**.

### 3.6 Expression profiles of FtC3H genes

**Figure 6:**
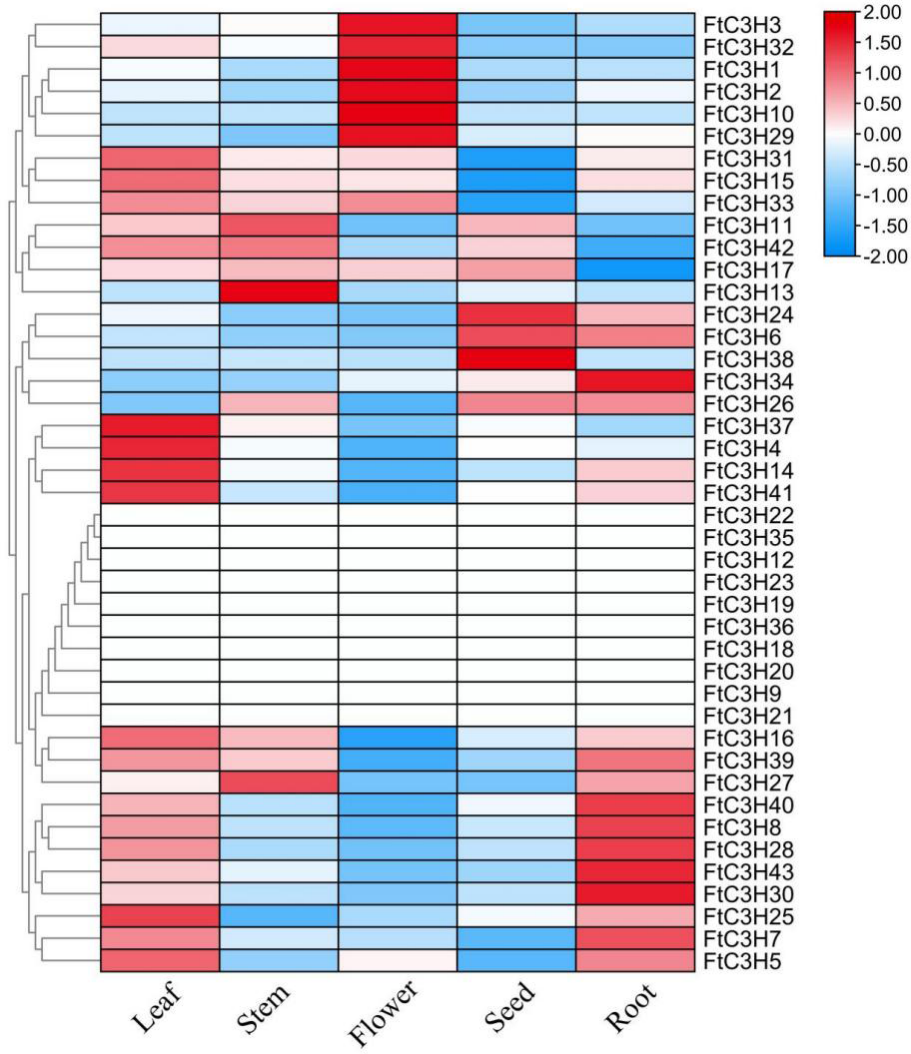
Tissue-specific expression profiles of FtC3H genes in Tartary buckwheat. The expression values were normalized for each row to visualize relative changes across different tissues. The color scale represents the relative expression levels, where red indicates high expression and blue indicates low expression.

To investigate the potential functions of the FtC3H gene family in different tissues of Tartary buckwheat, we analyzed their expression profiles in leaves, stems, flowers, seeds, and roots. The heatmap analysis revealed distinct tissue-specific expression patterns among the family members (**Figure 6**). Multiple genes, including *FtC3H3*, *FtC3H32*, *FtC3H1*, *FtC3H2*, *FtC3H10*, and *FtC3H29*, exhibited exceptionally high expression levels in flowers, implying their crucial roles in floral development or reproductive processes. In contrast, *FtC3H7*, *FtC3H25*, *FtC3H30*, *FtC3H8*, *FtC3H28*, *FtC3H43*, *FtC3H40*, and *FtC3H5* were predominantly expressed in roots, while *FtC3H13*, *FtC3H27*, *FtC3H16*, *FtC3H31*, *FtC3H11*, and *FtC3H17* showed high enrichment in stems. Furthermore, certain genes, such as *FtC3H22*, *FtC3H35*, *FtC3H12*, *FtC3H19*, *FtC3H23*, *FtC3H36*, *FtC3H18*, *FtC3H20*, *FtC3H9*, and *FtC3H21*, maintained very low basal expression levels across all examined tissues, suggesting that they might be induced by specific environmental cues or function specifically in other unexamined developmental stages or tissues.

**Figure 7:**
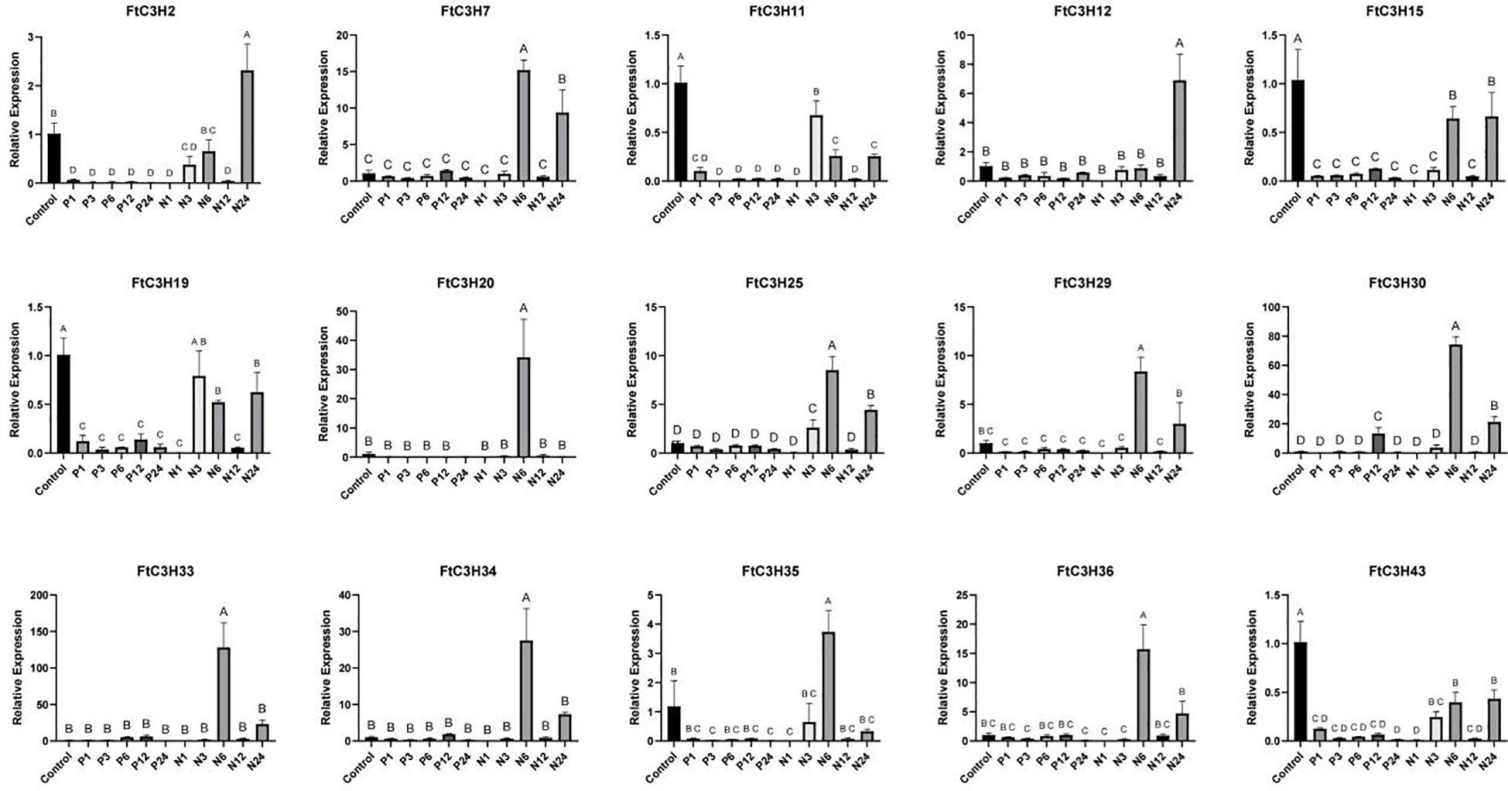
Expression analysis of FtC3H genes under salt (NaCl) and drought (PEG) stresses. Different uppercase letters (e.g., A, B, C) above the bars indicate significant differences among groups (P < 0.05).

RT-qPCR analysis demonstrated that salt stress acts as a potent inducer for the FtC3H gene family (**Figure 7**). As shown in the expression profiles, a large proportion of genes (including *FtC3H7*, *FtC3H12*, *FtC3H20*, *FtC3H25*, *FtC3H29*, *FtC3H30*, *FtC3H33*, etc.) were strongly induced by NaCl treatment. The peak expression typically occurred at 6 hours post-treatment, followed by a decline, which is characteristic of early-response transcription factors. The magnitude of induction was substantial, with *FtC3H33* exhibiting the maximum upregulation at 6 hours post-treatment by more than 100-fold, highlighting its potential as a master regulator in the salt stress signaling pathway. Conversely, the FtC3H genes appear to be less responsive to osmotic stress induced by PEG. Unlike the widespread upregulation observed under salinity, PEG treatment resulted in the suppression or negligible alteration of most FtC3H transcripts. Only a few exceptions were observed, such as *FtC3H19*, *FtC3H11*, and *FtC3H35*, which showed mild fluctuations under PEG treatment, but the magnitude of these changes was far less pronounced than the induction seen under salt stress. Notably, three genes, *FtC3H15*, *FtC3H19* and *FtC3H43*, showed the opposite pattern: they were highly expressed under control conditions but significantly downregulated under both NaCl and PEG treatments, suggesting potential negative regulatory roles in stress responses. This distinct expression pattern suggests that the FtC3H family in Tartary buckwheat has evolved to play a more specialized role in salt tolerance rather than general osmotic stress adaptation.

The transcriptional levels of 15 FtC3H genes are shown under control, PEG treatment (P1, P3, P6, P12, P24), and NaCl treatment (N1, N3, N6, N12, N24) conditions. Error bars represent the standard deviation (SD) of three replicates. Different letters indicate significant differences among groups (P < 0.05).

## 4 Discussion

Zinc finger motifs were first identified in the *Xenopus laevis* transcription factor IIIA. Since then, zinc finger proteins have been recognized as one of the most abundant protein families in eukaryotic genomes (Miller et al., 1985). In plants, zinc finger proteins serve as effective regulators of growth, development, and stress responses (Bogamuwa and Jang, 2014). Among them, C3H zinc finger proteins play pivotal roles in transcriptional regulation and protein-protein interactions (Han et al., 2021). To date, the C3H gene family has been extensively studied in various plants, including Brassica (Pi et al., 2018), poplar (Chai et al., 2012), and chickpea (Pradhan et al., 2017); however, research regarding this family in buckwheat remains limited. To understand how FtC3H proteins mediate stress signaling and developmental control at the molecular level, we identified and characterized this family in the buckwheat genome and examined their evolutionary relationships, interaction networks, and expression dynamics.

The C3H family has undergone a complex evolutionary process, becoming one of the largest gene families in plants (Chai et al., 2012; Wang et al., 2008). In this study, we identified 43 FtC3H genes. Genome-wide analyses revealed 68 and 67 C3H genes in Arabidopsis and rice (Wang et al., 2008), with an update reporting 73 in rice (Wang et al., 2025). Tomato has 47 (Li et al., 2025), soybean 140 (Alam et al., 2026). Other species include 40 in pineapple (Yang et al., 2025), 56 in mulberry (Chen et al., 2025), and 183 across four cotton species (Sun et al., 2025), indicating substantial variation in C3H family size across plants. Physicochemical property analysis revealed significant variations in protein length, molecular weight, instability index, theoretical isoelectric point, and aliphatic index among buckwheat C3H genes; however, all members were characterized as hydrophilic proteins. Subcellular localization predicted that FtC3H proteins are located in the nucleus, a feature shared by C3H proteins in mulberry (Chen et al., 2025), pineapple (Yang et al., 2025), and melon (Zheng et al., 2025), indicating their primary role as transcriptional regulators. Chromosomal localization indicated an uneven distribution of FtC3H genes across the eight chromosomes. Based on the phylogenetic trees of Arabidopsis and rice, the buckwheat C3H members were classified into six groups (Wang et al., 2008). This grouping reflects conserved evolutionary divergence within the C3H family, as similar clustering has been reported in cotton (Sun et al., 2025), pineapple (Yang et al., 2025), and melon (Zheng et al., 2025).Multiple sequence alignment revealed that the C-X8-C-X5-C-X3-H motif is abundant in FtC3H genes, a motif configuration also predominant in *Brassica napus* and *Brassica rapa* (Pi et al., 2021), suggesting it represents the ancestral form of plant C3H motifs.

Gene duplication is a primary driver of genomic and genetic system evolution (Tang et al., 2023). Synteny analysis in this study identified only four pairs of segmental duplication genes for FtC3H, with no tandem duplication pairs detected. Similarly, no tandem duplication pairs were found in the C3H gene families of pepper (Tang et al., 2023), and segmental duplication was also reported as the primary expansion mechanism for theC3H family in cucumber (Uddin et al., 2025). This suggests that segmental duplication may play a crucial role in the expansion of the C3H gene family. Consistent with this, analysis of duplicated gene pairs in the carrotC3H family showed that all 11 duplicated pairs underwent purifying selection (Saleem et al., 2025), and similar purifying selection (Ka/Ks < 1) was observed in the mulberryC3H family (Chen et al., 2025). The presence of 15–18 conserved syntenic gene pairs between Tartary buckwheat and the three reference species suggests that a core set of C3H genes was already established before the divergence of these lineages and has been maintained under purifying selection.

Gene structure analysis showed that five members (11.63%) of the buckwheat C3H family possess only a single exon. Intronless C3H genes have also been observed in other plant species. In mulberry, five A family members were identified as intronless, suggesting potential for rapid transcriptional responses in stress or development (Chen et al., 2025). In maize, the C3H IX subfamily contains 26 genes that are completely intronless (Chen et al., 2015). In barley, members of the C3H XI subfamily are also predominantly intronless (Ai et al., 2022). More broadly, intronless genes constitute a substantial fraction of plant genomes, accounting for 19.9% of rice genes and 21.7% of Arabidopsis genes, and many of them are evolutionarily conserved across the three domains of life (Jain et al., 2007). The presence of intronless C3H members in diverse angiosperms, together with their high conservation, supports the notion that this structural feature is evolutionarily conserved within the C3H gene family. Studies suggest that intronless genes may be more conserved during plant evolution, leading us to speculate that these five FtC3H genes are highly conserved (Jain et al., 2007).

C3H zinc finger proteins are classified into tandem C3H-type zinc finger (TZF) proteins and non-TZF proteins based on the number and distribution of C3H motifs (Pi et al., 2021). TZF proteins contain only two tandem C3H-type zinc finger motifs, whereas non-TZF proteins possess one or more than two motifs (Seok et al., 2018). Four FtC3H proteins (FtC3H8, FtC3H25, FtC3H29, and FtC3H32) were classified as TZF proteins, typically characterized by two C3H-type zinc finger motifs, classifying them as tandem C3H-type zinc finger proteins. Highly conserved plant TZF proteins are known regulators of plant growth, hormone responses, and stress reactions (Jang, 2016). For instance, the rice TZF gene *OsC3H10* is involved in regulating drought resistance (Han et al., 2021). Additionally, rice *OsTZF7* functions through the ABA signaling pathway to positively regulate drought adaptation (Guo et al., 2022). Collectively, the identification of TZF proteins within the FtC3H family, together with their conserved domain architecture and the functional precedents established in model species (Han et al., 2021; Guo et al., 2022), suggests that *FtC3H8*, *FtC3H25*, *FtC3H29*, and *FtC3H32* are strong candidates for conferring stress tolerance in buckwheat, acting potentially through ABA-dependent signaling pathways.

Regarding conserved motifs, the majority of FtC3H members contain at least two conserved motifs. The widespread distribution of Motif 1 across FtC3H members suggests its strong evolutionary conservation within the buckwheat C3H family. Overall, while exon-intron structures and conserved motifs vary between different subgroups, they remain similar within the same subgroup. The similarities within subgroups and specificities between subgroups suggest that genes within the same subgroup may share similar functions, while those in different subgroups may perform distinct functions (Tang et al., 2023).

Plant C3H zinc finger proteins interact with other proteins via their zinc finger domains, forming regulatory networks that coordinate stress and development (Bogamuwa and Jang, 2016). Using the Arabidopsis interaction network, we predicted potential interactions among FtC3H proteins. FtC3H23 was identified as a hub protein (interacting with 16 other FtC3H members), suggesting it integrates multiple stress signals, whereas FtC3H15 and FtC3H20, with only one partner each, may play peripheral roles. Using the C3H12 binding motif (MA1756.2), a large set of potential target genes was identified in the buckwheat genome. GO enrichment analysis showed these targets are involved in cellular and metabolic processes, indicating that FtC3H transcription factors broadly regulate growth and development.

*Cis*-acting elements in promoter regions are key binding sites for transcription factors, regulating gene expression in various biological processes (Hernandez-Garcia and Finer, 2014). This study identified multiple classes of *cis*-acting elements in the FtC3H promoter regions, categorized into hormone response, development regulation, and stress response. Regarding hormone regulation, Abscisic Acid (ABA) is a key messenger in plant responses to abiotic stress. ABA-responsive elements (ABRE) were detected in most FtC3H genes, with some containing multiple copies. Furthermore, many *FtC3H* genes contain MeJA-responsive elements, and 17 contain GA-responsive elements. In Arabidopsis, overexpression of *AtTZF1*, *AtTZF4*, *AtTZF5*, and *AtTZF6* resulted in phenotypes characterized by enhanced ABA function and reduced GA function, while loss-of-function mutants were more sensitive to salt, drought, and cold stresses (Lin et al., 2010; Bogamuwa and Jang, 2016). Similarly, PvC3H72 enhances cold tolerance in transgenic switchgrass by regulating the ICE1-CBF-COR complex and ABA signaling pathways (Xie et al., 2019), and *MdC3H49* from apple positively regulates drought tolerance (Zhang et al., 2026). Thus, FtC3H genes likely play crucial roles in hormone-mediated abiotic stress responses in buckwheat. In terms of growth and development, MSA-like elements were found in buckwheat C3H genes, which are known to regulate B-type cyclin expression in a plant-specific manner (Ito, 2000), suggesting that the FtC3H family may participate in cell cycle regulation. Regarding stress response, numerous elements related to anaerobic, low-temperature, and drought stresses were identified, with anaerobic-responsive elements being the most widely distributed, followed by low-temperature- and drought-responsive elements, indicating that FtC3H genes likely function in abiotic stress responses, as similarly observed in barley (Ai et al., 2022).

Notably, although the promoters of FtC3H genes commonly contain ABRE elements, this family exhibits general insensitivity or repression under PEG-simulated drought stress, in sharp contrast to the strong induction observed under salt stress. Our RT-qPCR data showed that most FtC3H genes were rapidly and highly upregulated by salt stress; for instance, *FtC3H33* peaked at 6 hours of salt treatment, suggesting it may act as a master regulator of salt tolerance. This widespread upregulation aligns closely with the enrichment of ABRE in the promoter regions, as ABRE serves as a core cis-acting element in the ABA signaling pathway that drives stress-inducible expression (Narusaka et al., 2003). The differential response between salt and PEG treatments suggests that activation of FtC3H genes may depend not only on ABRE but also on salt-stress-specific signals, such as Na^+^-induced membrane potential changes, SOS pathway activation, or specific ROS signals (Zhu et al., 2025). In contrast, the osmotic stress alone induced by PEG may not effectively mobilize these upstream signals and might even suppress FtC3H expression through unknown negative regulatory mechanisms. This pattern is consistent with observations in other species: the Arabidopsis non-TZF gene *AtC3H3* enhances salt tolerance without improving drought resistance (Seok et al., 2024), and multiple soybean C3H genes containing ABRE-rich promoters are significantly induced by salt stress (Alam et al., 2026). Taken together, these results indicate that during the evolution of tartary buckwheat, the FtC3H family has become functionally specialized as an early regulator of salt stress response rather than a general responder to osmotic stress.

## 5 Conclusion

This study presents the first genome-wide characterization of the C3H zinc finger protein family in Tartary buckwheat (*F. tataricum*). A total of 43 FtC3H genes were identified, which expanded mainly through segmental duplication under strong purifying selection. Phylogenetic and structural analyses revealed six conserved subgroups with distinct motif and exon-intron organizations. Promoter regions of FtC3H genes are highly enriched in ABRE and other stress-related cis-elements, linking them to ABA-dependent stress signaling. Notably, expression profiling uncovered a functional specialization: most FtC3H genes were strongly and rapidly induced by salt stress—exemplified by >100-fold upregulation of *FtC3H33*—but showed little or no response to PEG-simulated drought. This distinct regulatory pattern suggests that the FtC3H family in buckwheat has evolved primarily toward salt tolerance rather than general osmotic adaptation. Our findings provide a solid foundation for understanding C3H-mediated stress responses in Polygonaceae and suggest *FtC3H33* as a promising candidate for genetic improvement of salt tolerance in buckwheat and other crops.

## Data Availability

The original contributions presented in the study are included in the article/**Supplementary Material**. Further inquiries can be directed to the corresponding author.
