# Supplementary figures and images for: The C3H zinc finger gene family in tartary buckwheat: genome-wide characterization, evolution, and a specialized role in salt stress response
Source: Front Plant Sci. 2026 Jun 22;17:1865239. doi: 10.3389/fpls.2026.1865239 (PMC13333739; doi:10.3389/fpls.2026.1865239)

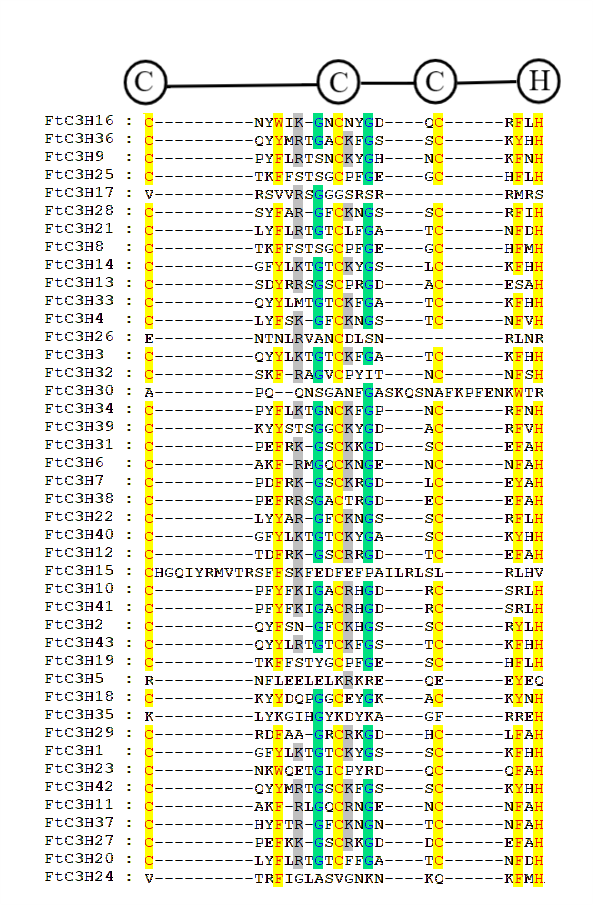

Supplement: Supplementary file 1 [file Image1.png]

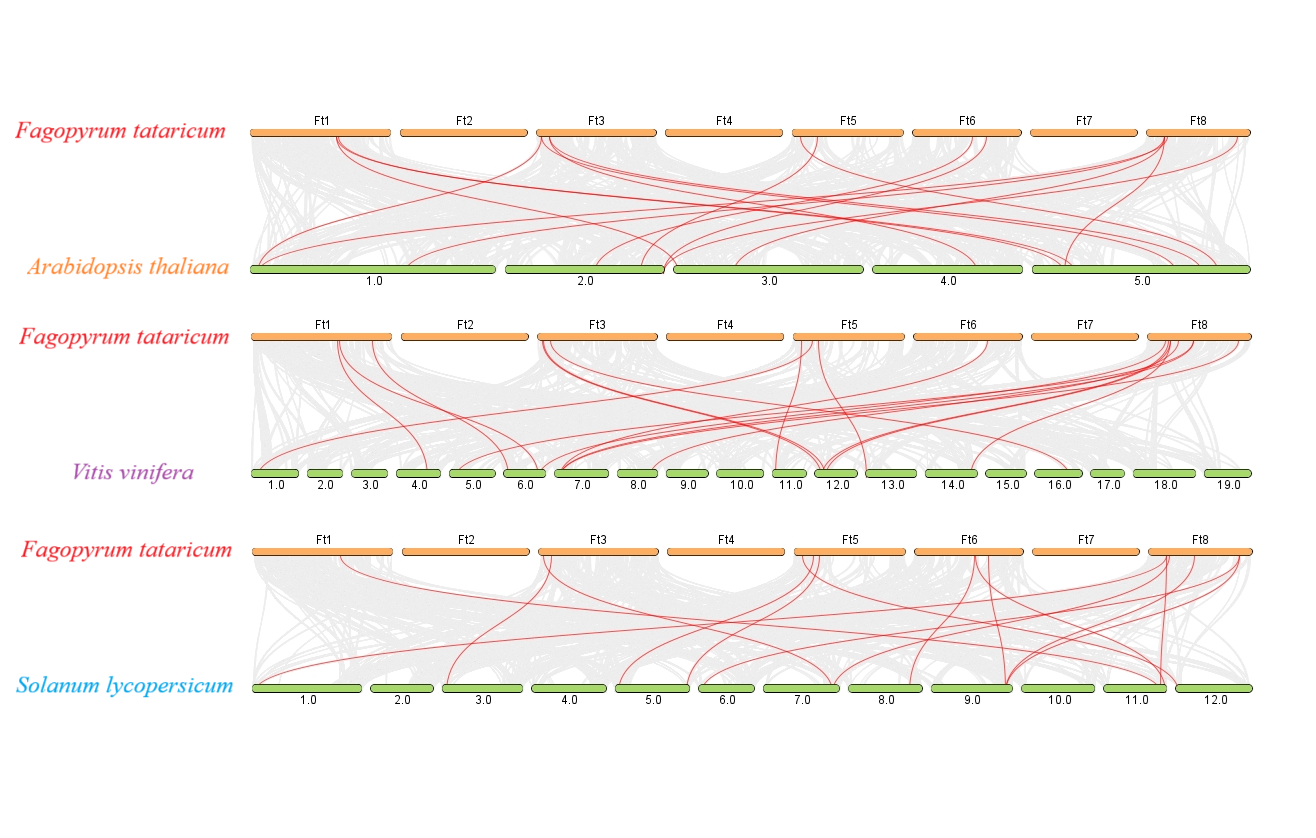

Supplement: Supplementary file 2 [file Image2.png]
